# The ACHIEVE Trial: Lessons Learned From Nesting a Randomized Controlled Trial Within an Observational Cohort Study

**DOI:** 10.1093/geroni/igaa057.2950

**Published:** 2020-12-16

**Authors:** Jennifer Deal, Nicholas Reed, David Couper, Kathleen Hayden, Thomas Mosley, Jim Pankow, Frank Lin, Josef Coresh

**Affiliations:** 1 Johns Hopkins Bloomberg School of Public Health, Baltimore, Maryland, United States; 2 Johns Hopkins University, Baltimore, Maryland, United States; 3 University of North Carolina at Chapel Hill, Chapel Hill, North Carolina, United States; 4 Wake Forest School of Medicine, Winston-Salem, North Carolina, United States; 5 University of Mississippi Medical Center, Jackson, Mississippi, United States; 6 University of Minnesota, Minneapolis, Minnesota, United States; 7 Johns Hopkins University School of Medicine, Baltimore, Maryland, United States

## Abstract

Hearing impairment in older adults is linked to accelerated cognitive decline and a 94% increased risk of incident dementia in population-based observational studies. Whether hearing treatment can delay cognitive decline is unknown but could have substantial clinical and public health impact. The NIH-funded ACHIEVE randomized controlled trial of 977 older adults aged 70-84 years with untreated mild-to-moderate hearing loss, is testing the efficacy of hearing treatment versus health education on cognitive decline over 3 years in community-dwelling older adults (Clinicaltrials.gov Identifier: NCT03243422.) This presentation will describe lessons learned from ACHIEVE’s unique study design. ACHIEVE is nested within a large, well-characterized multicenter observational study, the Atherosclerosis Risk in Communities Study. Such nesting within an observational study maximizes both operational and scientific efficiency. With trial results expected in 2022, this presentation will focus on the benefits gained in design and recruitment/retention, including dedicated study staff, well-established protocols, and established study staff-participant relationships. Part of a symposium sponsored by Sensory Health Interest Group.

